# Diaqua­bis­(5-carb­oxy-2-propyl-1*H*-imidazole-4-carboxyl­ato-κ^2^ *N* ^3^,*O* ^4^)manganese(II) 3.5-hydrate

**DOI:** 10.1107/S1600536810031612

**Published:** 2010-08-18

**Authors:** Shi-Jie Li, Dong-Liang Miao, Wen-Dong Song, Shi-Hong Li, Jian-Bin Yan

**Affiliations:** a College of Food Science and Technology, Guangdong Ocean University, Zhanjiang 524088, People’s Republic of China; b College of Science, Guangdong Ocean University, Zhanjiang 524088, People’s Republic of China; c College of Medical Laboratory, Hebei North University, Zhangjiakou 075000, People’s Republic of China

## Abstract

In the title complex, [Mn(C_8_H_9_N_2_O_4_)_2_(H_2_O)_2_]·3.5H_2_O, the Mn^II^ cation is six-coordinated by two *N*,*O*-bidentate H_2_pimda^−^ ligands (H_2_pimda^−^ = 5-carb­oxy-2-propyl-1*H*-imidazole-4-carboxyl­ate) and two water mol­ecules in a distorted octa­hedral environment. The complete solid-state structure can be described as a three-dimensional supra­molecular framework stabilized by a wide range of O—H⋯O and N—H⋯O hydrogen bonds. The propyl groups of H_2_pimda^−^ are disordered over two sets of sites with refined occupancies of 0.759 (5):0.241 (5) and 0.545 (7):0.455 (7).

## Related literature

For our previous structural studies of complexes with H_2_pimda^−^, see: Yan *et al.* (2010 ▶); Li *et al.* (2010 ▶); Song *et al.*(2010 ▶); He *et al.* (2010 ▶); Fan *et al.* (2010 ▶).
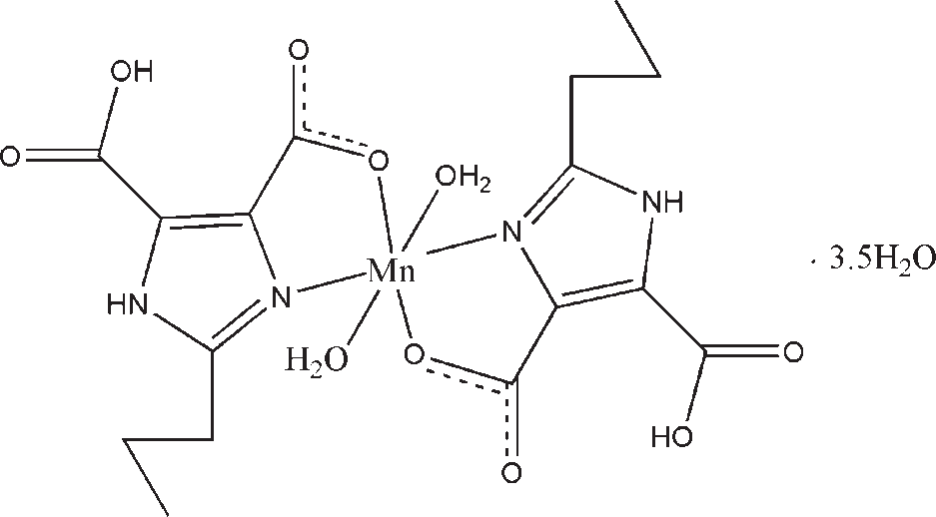


## Experimental

### 

#### Crystal data

[Mn(C_8_H_9_N_2_O_4_)_2_(H_2_O)_2_]·3.5H_2_O
*M* *_r_* = 548.37
Triclinic, 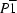

*a* = 10.609 (6) Å
*b* = 10.649 (6) Å
*c* = 11.424 (7) Å
α = 82.748 (8)°
β = 82.544 (7)°
γ = 86.857 (7)°
*V* = 1268.5 (13) Å^3^
*Z* = 2
Mo *K*α radiation
μ = 0.59 mm^−1^
*T* = 296 K
0.31 × 0.26 × 0.21 mm

#### Data collection

Bruker APEXII area-detector diffractometer
Absorption correction: multi-scan (*SADABS*; Bruker, 2004 ▶) *T*_min_ = 0.838, *T*_max_ = 0.886
6656 measured reflections
4508 independent reflections
2551 reflections with *I* > 2σ(*I*)
*R*_int_ = 0.035

#### Refinement

*R*[*F* ^2^ > 2σ(*F* ^2^)] = 0.059
*wR*(*F* ^2^) = 0.152
*S* = 1.00
4508 reflections
342 parameters
5 restraints
H-atom parameters constrained
Δρ_max_ = 0.32 e Å^−3^
Δρ_min_ = −0.33 e Å^−3^

### 

Data collection: *APEX2* (Bruker, 2004 ▶); cell refinement: *SAINT* (Bruker, 2004 ▶); data reduction: *SAINT*; program(s) used to solve structure: *SHELXS97* (Sheldrick, 2008 ▶); program(s) used to refine structure: *SHELXL97* (Sheldrick, 2008 ▶); molecular graphics: *SHELXTL* (Sheldrick, 2008 ▶); software used to prepare material for publication: *SHELXL97*.

## Supplementary Material

Crystal structure: contains datablocks I, global. DOI: 10.1107/S1600536810031612/jh2190sup1.cif

Structure factors: contains datablocks I. DOI: 10.1107/S1600536810031612/jh2190Isup2.hkl

Additional supplementary materials:  crystallographic information; 3D view; checkCIF report

## Figures and Tables

**Table 1:** Hydrogen-bond geometry (Å, °)

*D*—H⋯*A*	*D*—H	H⋯*A*	*D*⋯*A*	*D*—H⋯*A*
O1—H1⋯O4	0.82	1.70	2.507 (5)	167
O7—H6⋯O6	0.82	1.64	2.462 (4)	175
N2—H2⋯O4*W*	0.88	1.88	2.762 (6)	179
N4—H4⋯O6*W*	0.87	1.90	2.758 (5)	166
O1*W*—H1*W*⋯O5*W*^i^	0.85	2.25	2.667 (4)	110
O1*W*—H2*W*⋯O8^ii^	0.85	1.89	2.724 (4)	168
O2*W*—H3*W*⋯O8^iii^	0.85	2.09	2.878 (4)	153
O2*W*—H4*W*⋯O2^iv^	0.85	1.97	2.791 (4)	163
O3*W*—H5*W*⋯O3*W*^v^	0.85	1.48	2.149 (12)	133
O3*W*—H5*W*⋯O3^iv^	0.85	2.21	2.811 (6)	128
O3*W*—H6*W*⋯O3^vi^	0.86	1.98	2.793 (7)	157
O4*W*—H7*W*⋯O3*W*	0.85	1.82	2.646 (7)	165
O4*W*—H8*W*⋯O7^vii^	0.85	2.05	2.897 (5)	176
O5*W*—H9*W*⋯O5^vii^	0.85	2.09	2.885 (4)	156
O5*W*—H9*W*⋯O6^vii^	0.85	2.59	3.266 (5)	137
O5*W*—H10*W*⋯O4^viii^	0.85	1.97	2.804 (4)	166
O6*W*—H11*W*⋯O3*W*^viii^	0.85	1.86	2.674 (7)	160
O6*W*—H12*W*⋯O5*W*^ix^	0.85	2.26	2.880 (6)	130

Symmetry codes: (i) 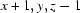; (ii) 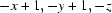; (iii) 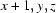; (iv) 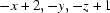; (v) 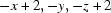; (vi) 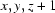; (vii) 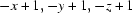; (viii) 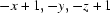; (ix) 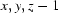.

**Fig. 1.**
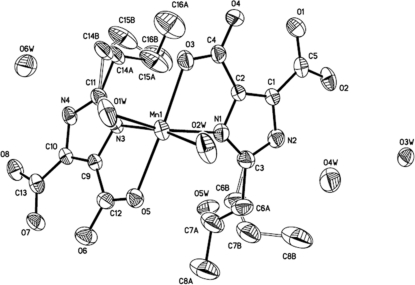
The structure of the title compound, showing the atomic numbering scheme. Non-H atoms are shown with 30% probability displacement ellipsoids.

## supplementary crystallographic information

### Comment

There is considerable interest in the design and synthesis of metal-organic frameworks (MOFs) due to their potential applications in conductivity, luminescence, catalysis, magnetism and sensors as well as fascinating architectures and topologies. 2-propyl-1*H*-imidazole-4,5-carboxylate(H_3_pimda) ligand as one derivative of H_3_IDC with efficient N,*O*-donors has been used to obtain new metal-organic complexes by our research group, such as poly[diaquabis(5-carboxy-2-propyl-1*H*-imidazole-4-carboxylato-k^3^ N^3^, O^4^,*O*^5^)calcium(II)](Song *et al.*, 2010), [diaquabis(5-carboxy-2-propyl-1*H*-imidazole-4-carboxylato-k^2^N^3^,*O*^4^) manganese(II)]*N*,*N*-dimethylformamide(Yan *et al.*, 2010), [Diaquabis(5-carboxy-2-propyl-1*H*-imidazole-4-carboxylato-k^2^*N*^3^,*O*^4^)nickle(II)]*N*,*N*-dimethylformamide disolvate(Li *et al.*, 2010), Diaquabis(4-carboxy-2-propyl-1*H*-imidazole-5-carboxylato- k^2^N^3^,*O*^4^)copper(II) *N*,*N*-dimethylformamide disolvate(He *et al.*, 2010) and Diaquabis(5-carboxy-2-propyl-1*H*-imidazole- 4-carboxylato-k^2^N^3^,*O*^4^)nickle(II) tetrahedrate(Fan *et al.*, 2010). In this paper, we report the structure of a new Mn(II) complex obtained under hydrothermal conditions.

As illustrated in figure 1, the title complex molecule is isomorphous with Ni(II) analog(Fan *et al.*, 2010). Similar structural description applies to the present isomorphous complex. The Mn^II^ is six-coordinated by two N,*O*-bidentate H_3_pimda anions and two water molecules, and exhibits a distorted octahedral geometry. The carboxylic acid ligand bears a formal charge of -1, and the free carboxylate atoms O1 and O4, O6 and O7 form intramolecular hydrogen bonds, respectively. The dihedral angle between the two imidazole rings is 77.2 (8) %A. In the crystal structure, the three-dimensional supramolecular framework is stabilized by extensive O—H···O and N—H···O hydrogen bonds involving the free water molecules, the coordinated water molecules, the carboxy O atoms and the protonated N atoms of H_3_pimda. The propyl groups of H_3_pimda are disordered over two sets of sites with refined occupiencies of 0.759 (5):0.241 (5) and 0.545 (7):0.455 (7).

### Experimental

A mixture of Mncl_2_ (0.5 mmol, 0.06 g) and 2-propyl-1*H*-imidazole-4,5-dicarboxylic acid(0.5 mmol, 0.99 g) in 15 ml of H_2_O solution was sealed in an autoclave equipped with a Teflon liner (20 ml) and then heated at 433k for 4 days. Crystals of the title compound were obtained by slow evaporation of the solvent at room temperature.

### Refinement

Water H atoms were located in a difference Fourier map and were allowed to ride on the parent atom, with *U*_iso_(H) = 1.5*U*_eq_(O). Carboxyl H atoms were located in a difference map and refined with distance restraints, *U*_iso_(H) = 1.5*U*_eq_(O). Other H atoms were placed at calculated positions and were treated as riding on parent atoms with C—H = 0.96 (methyl), 0.97 (methylene) and N—H = 0.86 Å, *U*_iso_(H) = 1.2 or 1.5*U*_eq_(C,*N*). The propyl groups of H_3_pimda are disordered over two sites with refined occupancies of 0.759 (5):0.241 (5) and 0.545 (7):0.455 (7). C—C distance restraints of disordered components were applied. The O3W water molecule is located close to an inversion center, its occupancy factor was refined to 0.49 (1) and was fixed as 0.5 at the final refinements.

### Crystal data

**Table:**

[Mn(C_8_H_9_N_2_O_4_)_2_(H_2_O)_2_]·3.5H_2_O	*Z* = 2
*M**_r_* = 548.37	*F*(000) = 572
Triclinic, *P*1	*D*_x_ = 1.436 Mg m^−^^3^
Hall symbol: -P 1	Mo *K*α radiation, λ = 0.71073 Å
*a* = 10.609 (6) Å	Cell parameters from 3600 reflections
*b* = 10.649 (6) Å	θ = 1.4–25.0°
*c* = 11.424 (7) Å	µ = 0.59 mm^−^^1^
α = 82.748 (8)°	*T* = 296 K
β = 82.544 (7)°	Block, colorless
γ = 86.857 (7)°	0.31 × 0.26 × 0.21 mm
*V* = 1268.5 (13) Å^3^	

### Data collection

**Table:**

Bruker APEXII area-detector diffractometer	4508 independent reflections
Radiation source: fine-focus sealed tube	2551 reflections with *I* > 2σ(*I*)
graphite	*R*_int_ = 0.035
φ and ω scan	θ_max_ = 25.2°, θ_min_ = 1.8°
Absorption correction: multi-scan (*SADABS*; Bruker, 2004)	*h* = −12→12
*T*_min_ = 0.838, *T*_max_ = 0.886	*k* = −12→12
6656 measured reflections	*l* = −13→10

### Refinement

**Table:**

Refinement on *F*^2^	Primary atom site location: structure-invariant direct methods
Least-squares matrix: full	Secondary atom site location: difference Fourier map
*R*[*F*^2^ > 2σ(*F*^2^)] = 0.059	Hydrogen site location: inferred from neighbouring sites
*wR*(*F*^2^) = 0.152	H-atom parameters constrained
*S* = 1.00	*w* = 1/[σ^2^(*F*_o_^2^) + (0.0585*P*)^2^ + 0.2346*P*] where *P* = (*F*_o_^2^ + 2*F*_c_^2^)/3
4508 reflections	(Δ/σ)_max_ = 0.009
342 parameters	Δρ_max_ = 0.32 e Å^−^^3^
5 restraints	Δρ_min_ = −0.33 e Å^−^^3^

### Special details

**Table:**

Geometry. All e.s.d.'s (except the e.s.d. in the dihedral angle between two l.s. planes) are estimated using the full covariance matrix. The cell e.s.d.'s are taken into account individually in the estimation of e.s.d.'s in distances, angles and torsion angles; correlations between e.s.d.'s in cell parameters are only used when they are defined by crystal symmetry. An approximate (isotropic) treatment of cell e.s.d.'s is used for estimating e.s.d.'s involving l.s. planes.
Refinement. Refinement of *F*^2^ against ALL reflections. The weighted *R*-factor *wR* and goodness of fit *S* are based on *F*^2^, conventional *R*-factors *R* are based on *F*, with *F* set to zero for negative *F*^2^. The threshold expression of *F*^2^ > σ(*F*^2^) is used only for calculating *R*-factors(gt) *etc*. and is not relevant to the choice of reflections for refinement. *R*-factors based on *F*^2^ are statistically about twice as large as those based on *F*, and *R*- factors based on ALL data will be even larger.

### Fractional atomic coordinates and isotropic or equivalent isotropic displacement parameters (Å^2^)

**Table:**

	*x*	*y*	*z*	*U*_iso_*/*U*_eq_	Occ. (<1)
Mn1	0.84879 (6)	0.29330 (6)	0.19294 (6)	0.0505 (2)	
O1	0.8746 (4)	−0.1883 (3)	0.5157 (3)	0.0718 (10)	
H1	0.8701	−0.1626	0.4455	0.108*	
O2	0.8374 (3)	−0.1110 (3)	0.6882 (3)	0.0724 (10)	
O3	0.8872 (3)	0.0868 (3)	0.1898 (3)	0.0582 (8)	
O4	0.8969 (3)	−0.1020 (3)	0.2997 (3)	0.0662 (9)	
O5	0.7711 (3)	0.4910 (3)	0.2083 (3)	0.0564 (8)	
O6	0.6057 (3)	0.6263 (3)	0.1848 (3)	0.0691 (10)	
O7	0.3863 (3)	0.6187 (3)	0.1382 (3)	0.0641 (9)	
H6	0.4581	0.6182	0.1577	0.096*	
O8	0.2555 (3)	0.4775 (3)	0.0983 (3)	0.0642 (9)	
N1	0.8290 (3)	0.2105 (3)	0.3841 (3)	0.0500 (9)	
N2	0.8112 (3)	0.1375 (4)	0.5753 (3)	0.0585 (10)	
H2	0.7991	0.1451	0.6516	0.070*	
N3	0.6401 (3)	0.2898 (3)	0.1720 (3)	0.0460 (9)	
N4	0.4436 (3)	0.2822 (3)	0.1290 (3)	0.0505 (9)	
H4	0.3776	0.2468	0.1111	0.061*	
C1	0.8355 (4)	0.0337 (4)	0.5152 (4)	0.0440 (10)	
C2	0.8473 (3)	0.0802 (4)	0.3970 (4)	0.0419 (10)	
C3	0.8087 (5)	0.2420 (4)	0.4943 (4)	0.0604 (13)	
C4	0.8790 (4)	0.0165 (4)	0.2877 (4)	0.0496 (11)	
C5	0.8483 (4)	−0.0942 (5)	0.5799 (5)	0.0554 (12)	
C6A	0.7996 (11)	0.3774 (11)	0.5220 (13)	0.084 (3)	0.759 (5)
H6A	0.8314	0.3810	0.5973	0.100*	0.759 (5)
H6B	0.8528	0.4288	0.4608	0.100*	0.759 (5)
C7A	0.6680 (8)	0.4301 (7)	0.5284 (8)	0.100 (3)	0.759 (5)
H7A	0.6169	0.3863	0.5964	0.120*	0.759 (5)
H7B	0.6321	0.4168	0.4573	0.120*	0.759 (5)
C8A	0.6635 (9)	0.5741 (7)	0.5402 (9)	0.137 (4)	0.759 (5)
H8A	0.5779	0.6013	0.5665	0.206*	0.759 (5)
H8B	0.6914	0.6202	0.4643	0.206*	0.759 (5)
H8C	0.7183	0.5897	0.5969	0.206*	0.759 (5)
C6B	0.747 (4)	0.358 (4)	0.541 (5)	0.084 (3)	0.241 (5)
H6C	0.6822	0.3936	0.4932	0.100*	0.241 (5)
H6D	0.7079	0.3376	0.6224	0.100*	0.241 (5)
C7B	0.846 (2)	0.452 (3)	0.538 (2)	0.100 (3)	0.241 (5)
H7C	0.8067	0.5350	0.5479	0.120*	0.241 (5)
H7D	0.8990	0.4584	0.4612	0.120*	0.241 (5)
C8B	0.929 (3)	0.410 (2)	0.638 (3)	0.137 (4)	0.241 (5)
H8D	0.9855	0.4767	0.6433	0.206*	0.241 (5)
H8E	0.9788	0.3351	0.6207	0.206*	0.241 (5)
H8F	0.8759	0.3933	0.7120	0.206*	0.241 (5)
C9	0.5827 (4)	0.4087 (4)	0.1688 (4)	0.0412 (10)	
C10	0.4601 (4)	0.4059 (4)	0.1419 (4)	0.0432 (10)	
C11	0.5528 (4)	0.2139 (4)	0.1468 (4)	0.0515 (11)	
C12	0.6575 (4)	0.5144 (4)	0.1884 (4)	0.0507 (11)	
C13	0.3590 (4)	0.5048 (4)	0.1255 (4)	0.0526 (12)	
C14A	0.559 (3)	0.0724 (6)	0.1680 (13)	0.061 (3)	0.545 (7)
H14A	0.6447	0.0413	0.1436	0.073*	0.545 (7)
H14B	0.5023	0.0391	0.1202	0.073*	0.545 (7)
C15A	0.5196 (17)	0.0242 (9)	0.3025 (12)	0.093 (4)	0.545 (7)
H15A	0.5725	0.0607	0.3516	0.111*	0.545 (7)
H15B	0.4315	0.0488	0.3263	0.111*	0.545 (7)
C16A	0.5371 (12)	−0.1179 (9)	0.3172 (12)	0.107 (4)	0.545 (7)
H16A	0.5074	−0.1508	0.3976	0.160*	0.545 (7)
H16B	0.6257	−0.1410	0.2996	0.160*	0.545 (7)
H16C	0.4894	−0.1524	0.2637	0.160*	0.545 (7)
C14B	0.578 (3)	0.0799 (8)	0.1201 (18)	0.061 (3)	0.455 (7)
H14C	0.6668	0.0560	0.1241	0.073*	0.455 (7)
H14D	0.5594	0.0731	0.0402	0.073*	0.455 (7)
C15B	0.4928 (12)	−0.0126 (12)	0.2116 (12)	0.093 (4)	0.455 (7)
H15C	0.4039	0.0123	0.2083	0.111*	0.455 (7)
H15D	0.5061	−0.0979	0.1897	0.111*	0.455 (7)
C16B	0.523 (2)	−0.0117 (16)	0.3355 (14)	0.107 (4)	0.455 (7)
H16D	0.5291	−0.0971	0.3736	0.160*	0.455 (7)
H16E	0.4576	0.0352	0.3793	0.160*	0.455 (7)
H16F	0.6032	0.0274	0.3332	0.160*	0.455 (7)
O1W	0.8931 (3)	0.3345 (3)	0.0048 (3)	0.0828 (11)	
H1W	0.9099	0.2672	−0.0280	0.124*	
H2W	0.8568	0.3968	−0.0336	0.124*	
O2W	1.0434 (3)	0.3326 (3)	0.2140 (3)	0.0849 (12)	
H3W	1.0867	0.3943	0.1787	0.127*	
H4W	1.0929	0.2699	0.2328	0.127*	
O3W	0.9127 (6)	0.0143 (5)	0.9610 (5)	0.0656 (17)	0.50
H5W	0.9930	0.0018	0.9532	0.098*	0.50
H6W	0.9068	0.0143	1.0364	0.098*	0.50
O4W	0.7778 (4)	0.1620 (4)	0.8153 (4)	0.1275 (17)	
H7W	0.8316	0.1163	0.8523	0.191*	
H8W	0.7326	0.2287	0.8273	0.191*	
O5W	0.1048 (3)	0.2730 (3)	0.8677 (3)	0.0828 (11)	
H9W	0.1543	0.3290	0.8309	0.124*	
H10W	0.1101	0.2120	0.8256	0.124*	
O6W	0.2622 (3)	0.1565 (4)	0.0418 (4)	0.1110 (15)	
H11W	0.2152	0.0928	0.0546	0.167*	
H12W	0.2063	0.2152	0.0292	0.167*	

### Atomic displacement parameters (Å^2^)

**Table:**

	*U*^11^	*U*^22^	*U*^33^	*U*^12^	*U*^13^	*U*^23^
Mn1	0.0448 (4)	0.0542 (5)	0.0498 (5)	0.0021 (3)	−0.0097 (3)	0.0064 (3)
O1	0.091 (2)	0.051 (2)	0.071 (3)	0.0000 (18)	−0.019 (2)	0.0070 (18)
O2	0.073 (2)	0.082 (2)	0.052 (2)	0.0119 (18)	−0.0074 (18)	0.0235 (18)
O3	0.065 (2)	0.062 (2)	0.044 (2)	0.0122 (16)	−0.0093 (16)	0.0003 (16)
O4	0.094 (2)	0.049 (2)	0.058 (2)	0.0116 (17)	−0.0190 (18)	−0.0090 (16)
O5	0.0510 (18)	0.0522 (19)	0.068 (2)	−0.0029 (14)	−0.0201 (16)	−0.0028 (16)
O6	0.064 (2)	0.050 (2)	0.096 (3)	0.0058 (16)	−0.0150 (19)	−0.0153 (18)
O7	0.0532 (19)	0.057 (2)	0.081 (3)	0.0147 (16)	−0.0147 (18)	−0.0039 (18)
O8	0.0415 (17)	0.072 (2)	0.074 (2)	0.0008 (16)	−0.0118 (16)	0.0119 (17)
N1	0.051 (2)	0.054 (2)	0.043 (2)	0.0040 (17)	−0.0077 (18)	−0.0020 (18)
N2	0.063 (2)	0.070 (3)	0.041 (2)	0.007 (2)	−0.0037 (19)	−0.008 (2)
N3	0.046 (2)	0.040 (2)	0.051 (2)	0.0014 (16)	−0.0115 (17)	0.0029 (16)
N4	0.0364 (19)	0.053 (2)	0.061 (3)	−0.0019 (17)	−0.0159 (17)	0.0055 (18)
C1	0.040 (2)	0.048 (3)	0.043 (3)	0.0023 (19)	−0.005 (2)	0.000 (2)
C2	0.039 (2)	0.042 (2)	0.044 (3)	0.0022 (18)	−0.008 (2)	−0.002 (2)
C3	0.078 (3)	0.053 (3)	0.049 (3)	0.005 (2)	−0.008 (3)	−0.004 (2)
C4	0.044 (2)	0.055 (3)	0.051 (3)	0.008 (2)	−0.013 (2)	−0.006 (2)
C5	0.047 (3)	0.061 (3)	0.055 (3)	−0.002 (2)	−0.013 (2)	0.010 (3)
C6A	0.125 (12)	0.063 (6)	0.060 (7)	0.014 (7)	−0.005 (8)	−0.013 (5)
C7A	0.122 (7)	0.068 (5)	0.106 (7)	0.015 (5)	−0.002 (5)	−0.013 (5)
C8A	0.177 (10)	0.076 (6)	0.154 (10)	0.023 (6)	0.001 (7)	−0.025 (6)
C6B	0.125 (12)	0.063 (6)	0.060 (7)	0.014 (7)	−0.005 (8)	−0.013 (5)
C7B	0.122 (7)	0.068 (5)	0.106 (7)	0.015 (5)	−0.002 (5)	−0.013 (5)
C8B	0.177 (10)	0.076 (6)	0.154 (10)	0.023 (6)	0.001 (7)	−0.025 (6)
C9	0.041 (2)	0.040 (2)	0.041 (3)	0.0010 (19)	−0.0066 (19)	0.0023 (19)
C10	0.043 (2)	0.041 (3)	0.042 (3)	0.0043 (19)	−0.0047 (19)	0.0032 (19)
C11	0.045 (2)	0.047 (3)	0.062 (3)	−0.002 (2)	−0.013 (2)	0.000 (2)
C12	0.054 (3)	0.046 (3)	0.052 (3)	−0.001 (2)	−0.007 (2)	−0.003 (2)
C13	0.047 (3)	0.057 (3)	0.048 (3)	0.002 (2)	0.001 (2)	0.008 (2)
C14A	0.058 (8)	0.042 (3)	0.082 (12)	0.002 (3)	−0.023 (10)	0.004 (4)
C15A	0.073 (6)	0.056 (6)	0.143 (12)	−0.005 (5)	−0.023 (7)	0.021 (6)
C16A	0.116 (8)	0.068 (6)	0.133 (9)	−0.005 (7)	−0.022 (7)	0.006 (7)
C14B	0.058 (8)	0.042 (3)	0.082 (12)	0.002 (3)	−0.023 (10)	0.004 (4)
C15B	0.073 (6)	0.056 (6)	0.143 (12)	−0.005 (5)	−0.023 (7)	0.021 (6)
C16B	0.116 (8)	0.068 (6)	0.133 (9)	−0.005 (7)	−0.022 (7)	0.006 (7)
O1W	0.078 (2)	0.097 (3)	0.060 (2)	0.036 (2)	−0.0015 (18)	0.0170 (19)
O2W	0.052 (2)	0.088 (3)	0.105 (3)	−0.0148 (18)	−0.0197 (19)	0.043 (2)
O3W	0.072 (4)	0.074 (4)	0.051 (4)	0.006 (3)	−0.013 (3)	−0.011 (3)
O4W	0.156 (4)	0.155 (4)	0.078 (3)	0.069 (3)	−0.035 (3)	−0.053 (3)
O5W	0.073 (2)	0.078 (2)	0.100 (3)	−0.0245 (19)	0.013 (2)	−0.035 (2)
O6W	0.099 (3)	0.108 (3)	0.144 (4)	0.006 (2)	−0.064 (3)	−0.038 (3)

### Geometric parameters (Å, °)

**Table:**

Mn1—O1W	2.134 (3)	C6B—H6D	0.9700
Mn1—O2W	2.178 (3)	C7B—C8B	1.540 (13)
Mn1—O3	2.218 (3)	C7B—H7C	0.9700
Mn1—N1	2.237 (4)	C7B—H7D	0.9700
Mn1—O5	2.238 (3)	C8B—H8D	0.9600
Mn1—N3	2.260 (3)	C8B—H8E	0.9600
O1—C5	1.312 (5)	C8B—H8F	0.9600
O1—H1	0.8200	C9—C10	1.377 (5)
O2—C5	1.219 (5)	C9—C12	1.468 (6)
O3—C4	1.261 (5)	C10—C13	1.474 (5)
O4—C4	1.259 (5)	C11—C14A	1.495 (7)
O5—C12	1.260 (5)	C11—C14B	1.499 (9)
O6—C12	1.283 (5)	C14A—C15A	1.567 (14)
O7—C13	1.292 (5)	C14A—H14A	0.9700
O7—H6	0.8200	C14A—H14B	0.9700
O8—C13	1.238 (5)	C15A—C16A	1.505 (11)
N1—C3	1.332 (5)	C15A—H15A	0.9700
N1—C2	1.382 (5)	C15A—H15B	0.9700
N2—C3	1.356 (5)	C16A—H16A	0.9600
N2—C1	1.369 (5)	C16A—H16B	0.9600
N2—H2	0.8771	C16A—H16C	0.9600
N3—C11	1.344 (5)	C14B—C15B	1.571 (14)
N3—C9	1.373 (5)	C14B—H14C	0.9700
N4—C11	1.358 (5)	C14B—H14D	0.9700
N4—C10	1.367 (5)	C15B—C16B	1.495 (12)
N4—H4	0.8708	C15B—H15C	0.9700
C1—C2	1.371 (6)	C15B—H15D	0.9700
C1—C5	1.474 (6)	C16B—H16D	0.9600
C2—C4	1.487 (6)	C16B—H16E	0.9600
C3—C6B	1.50 (5)	C16B—H16F	0.9600
C3—C6A	1.510 (12)	O1W—H1W	0.8500
C6A—C7A	1.472 (14)	O1W—H2W	0.8500
C6A—H6A	0.9700	O2W—H3W	0.8501
C6A—H6B	0.9700	O2W—H4W	0.8500
C7A—C8A	1.553 (9)	O3W—H5W	0.8499
C7A—H7A	0.9700	O3W—H6W	0.8551
C7A—H7B	0.9700	O4W—H7W	0.8500
C8A—H8A	0.9600	O4W—H8W	0.8500
C8A—H8B	0.9600	O5W—H9W	0.8500
C8A—H8C	0.9600	O5W—H10W	0.8500
C6B—C7B	1.482 (17)	O6W—H11W	0.8500
C6B—H6C	0.9700	O6W—H12W	0.8500
			
O1W—Mn1—O2W	89.56 (13)	C8B—C7B—H7C	109.8
O1W—Mn1—O3	93.19 (12)	C6B—C7B—H7D	109.8
O2W—Mn1—O3	94.68 (12)	C8B—C7B—H7D	109.8
O1W—Mn1—N1	167.13 (13)	H7C—C7B—H7D	108.3
O2W—Mn1—N1	86.66 (12)	C7B—C8B—H8D	109.5
O3—Mn1—N1	74.89 (12)	C7B—C8B—H8E	109.5
O1W—Mn1—O5	91.53 (12)	H8D—C8B—H8E	109.5
O2W—Mn1—O5	95.47 (12)	C7B—C8B—H8F	109.5
O3—Mn1—O5	168.84 (11)	H8D—C8B—H8F	109.5
N1—Mn1—O5	101.07 (12)	H8E—C8B—H8F	109.5
O1W—Mn1—N3	89.97 (12)	N3—C9—C10	110.4 (3)
O2W—Mn1—N3	169.81 (13)	N3—C9—C12	118.4 (3)
O3—Mn1—N3	95.51 (11)	C10—C9—C12	131.2 (4)
N1—Mn1—N3	95.91 (12)	N4—C10—C9	105.1 (3)
O5—Mn1—N3	74.37 (11)	N4—C10—C13	122.0 (4)
C5—O1—H1	109.5	C9—C10—C13	132.9 (4)
C4—O3—Mn1	118.1 (3)	N3—C11—N4	109.9 (4)
C12—O5—Mn1	116.7 (3)	N3—C11—C14A	125.3 (13)
C13—O7—H6	109.5	N4—C11—C14A	122.8 (12)
C3—N1—C2	105.4 (3)	N3—C11—C14B	125.4 (15)
C3—N1—Mn1	142.5 (3)	N4—C11—C14B	123.9 (15)
C2—N1—Mn1	112.1 (3)	C14A—C11—C14B	21.4 (9)
C3—N2—C1	108.2 (4)	O5—C12—O6	122.5 (4)
C3—N2—H2	120.0	O5—C12—C9	118.2 (4)
C1—N2—H2	131.8	O6—C12—C9	119.3 (4)
C11—N3—C9	105.7 (3)	O8—C13—O7	123.3 (4)
C11—N3—Mn1	141.8 (3)	O8—C13—C10	120.2 (4)
C9—N3—Mn1	111.8 (3)	O7—C13—C10	116.4 (4)
C11—N4—C10	108.9 (3)	C11—C14A—C15A	111.1 (7)
C11—N4—H4	121.3	C11—C14A—H14A	109.4
C10—N4—H4	129.8	C15A—C14A—H14A	109.4
N2—C1—C2	105.5 (4)	C11—C14A—H14B	109.4
N2—C1—C5	120.8 (4)	C15A—C14A—H14B	109.4
C2—C1—C5	133.7 (4)	H14A—C14A—H14B	108.0
C1—C2—N1	110.1 (4)	C16A—C15A—C14A	107.5 (10)
C1—C2—C4	131.7 (4)	C16A—C15A—H15A	110.2
N1—C2—C4	118.2 (4)	C14A—C15A—H15A	110.2
N1—C3—N2	110.8 (4)	C16A—C15A—H15B	110.2
N1—C3—C6B	131 (2)	C14A—C15A—H15B	110.2
N2—C3—C6B	115 (2)	H15A—C15A—H15B	108.5
N1—C3—C6A	123.2 (7)	C11—C14B—C15B	110.5 (12)
N2—C3—C6A	125.7 (7)	C11—C14B—H14C	109.5
C6B—C3—C6A	23.1 (13)	C15B—C14B—H14C	109.5
O4—C4—O3	125.2 (4)	C11—C14B—H14D	109.5
O4—C4—C2	118.1 (4)	C15B—C14B—H14D	109.5
O3—C4—C2	116.8 (4)	H14C—C14B—H14D	108.1
O2—C5—O1	121.7 (4)	C16B—C15B—C14B	111.5 (16)
O2—C5—C1	121.2 (5)	C16B—C15B—H15C	109.3
O1—C5—C1	117.0 (4)	C14B—C15B—H15C	109.3
C7A—C6A—C3	112.0 (8)	C16B—C15B—H15D	109.3
C7A—C6A—H6A	109.2	C14B—C15B—H15D	109.3
C3—C6A—H6A	109.2	H15C—C15B—H15D	108.0
C7A—C6A—H6B	109.2	C15B—C16B—H16D	109.5
C3—C6A—H6B	109.2	C15B—C16B—H16E	109.5
H6A—C6A—H6B	107.9	H16D—C16B—H16E	109.5
C6A—C7A—C8A	111.0 (8)	C15B—C16B—H16F	109.5
C6A—C7A—H7A	109.4	H16D—C16B—H16F	109.5
C8A—C7A—H7A	109.4	H16E—C16B—H16F	109.5
C6A—C7A—H7B	109.4	Mn1—O1W—H1W	111.4
C8A—C7A—H7B	109.4	Mn1—O1W—H2W	121.1
H7A—C7A—H7B	108.0	H1W—O1W—H2W	118.1
C7B—C6B—C3	109 (3)	Mn1—O2W—H3W	127.0
C7B—C6B—H6C	110.0	Mn1—O2W—H4W	117.6
C3—C6B—H6C	110.0	H3W—O2W—H4W	109.8
C7B—C6B—H6D	110.0	H5W—O3W—H6W	93.8
C3—C6B—H6D	110.0	H7W—O4W—H8W	135.3
H6C—C6B—H6D	108.3	H9W—O5W—H10W	106.9
C6B—C7B—C8B	109 (3)	H11W—O6W—H12W	99.7
C6B—C7B—H7C	109.8		

### Hydrogen-bond geometry (Å, °)

**Table:**

*D*—H···*A*	*D*—H	H···*A*	*D*···*A*	*D*—H···*A*
O1—H1···O4	0.82	1.70	2.507 (5)	167.
O7—H6···O6	0.82	1.64	2.462 (4)	175.
N2—H2···O4W	0.88	1.88	2.762 (6)	179.
N4—H4···O6W	0.87	1.90	2.758 (5)	166.
O1W—H1W···O5W^i^	0.85	2.25	2.667 (4)	110.
O1W—H2W···O8^ii^	0.85	1.89	2.724 (4)	168.
O2W—H3W···O8^iii^	0.85	2.09	2.878 (4)	153.
O2W—H4W···O2^iv^	0.85	1.97	2.791 (4)	163.
O3W—H5W···O3W^v^	0.85	1.48	2.149 (12)	133.
O3W—H5W···O3^iv^	0.85	2.21	2.811 (6)	128.
O3W—H6W···O3^vi^	0.86	1.98	2.793 (7)	157.
O4W—H7W···O3W	0.85	1.82	2.646 (7)	165.
O4W—H8W···O7^vii^	0.85	2.05	2.897 (5)	176.
O5W—H9W···O5^vii^	0.85	2.09	2.885 (4)	156.
O5W—H9W···O6^vii^	0.85	2.59	3.266 (5)	137.
O5W—H10W···O4^viii^	0.85	1.97	2.804 (4)	166.
O6W—H11W···O3W^viii^	0.85	1.86	2.674 (7)	160.
O6W—H12W···O5W^ix^	0.85	2.26	2.880 (6)	130.

Symmetry codes: (i) *x*+1, *y*, *z*−1; (ii) −*x*+1, −*y*+1, −*z*; (iii) *x*+1, *y*, *z*; (iv) −*x*+2, −*y*, −*z*+1; (v) −*x*+2, −*y*, −*z*+2; (vi) *x*, *y*, *z*+1; (vii) −*x*+1, −*y*+1, −*z*+1; (viii) −*x*+1, −*y*, −*z*+1; (ix) *x*, *y*, *z*−1.
